# 

**Published:** 1878-08-20

**Authors:** 


					﻿a
05
M
f’l
o
I—I
Q
a
2
—«
&
Z—s
L>
O
Si
P
o
"1
CO
H
a
O
co
. (—I
U
O
a
HH
rH
H
1""^
£<
a
<n
a
PU
a
co
<1
a
a
CL.
VOL. VIII.	AUGUST 20, 1878.	No. 8.
THE SOUTHER^
MEDICAL EECOED.
^ONTHLY jJoUF^NAL OF J^ACTICAL ^A-EDICINE.
~	/£»-GcO'\
W£X&J
Terms: 92 OO per annum in advance. Clubliates, 5 copies
88.00. Single copies 840 cents.
** QUICQVTD PRsECIPIES ESTO BREVIS,99
£
■—
e® I
o
o
rH
h-4
Q
H
t—i
O
co
>
s
o
H
HH
o
>
I—1
L.
I—I
I--
hn
LJ
CO
CO
o
h—<
2
H
W
U
ESTABLISHED IN 1870.
ATLANTA, GA.:
Sunny South Steam Publishing House.
Address R. C. WORD, M. D., Managing Editor, Atlanta, Ga.
				

